# Early full weight bearing is safe in open-wedge high tibial osteotomy: RSA analysis of postoperative stability compared to delayed weight bearing

**DOI:** 10.3109/17453671003619003

**Published:** 2010-04-06

**Authors:** Justus-Martijn Brinkman, Joan WH Luites, Ate B Wymenga, Ronald J van Heerwaarden

**Affiliations:** ^1^Limb Deformity Reconstruction Unit, Department of Orthopaedics; ^2^Department of Research, Development and Education, OrthoResearch Unit, Sint Maartenskliniek, Nijmegen & Woerdenthe Netherlands

## Abstract

**Background and purpose** In open-wedge, valgus osteotomy of the upper tibia, there are concerns regarding the initial stability and ability to retain the correction. Rehabilitation protocols vary depending on the osteotomy technique and the fixation method. Angle-stable implants offer superior initial stability. Early full weight bearing appears to be possible using these implants. In this prospective cohort study, we measured migration in open-wedge osteotomy in patients following an early full weight bearing protocol and compared the results to those from a historical cohort of open-wedge osteotomy patients who followed a standard protocol (full weight bearing after 6 weeks) using radiostereometry.

**Methods** 14 open-wedge osteotomies fixated with the angle-stable Tomofix implant were performed; patients were allowed full weight bearing as soon as pain and wound healing permitted. Radiostereometry was used to measure motion across the osteotomy at regular intervals. Improvement in pain and functional outcome were assessed postoperatively. The results were compared to those from a group of 23 patients who had undergone the same operation but had used a standard rehabilitation protocol.

**Results** There were no adverse effects because of the early full weight bearing protocol. There were no differences in motion at the osteotomy between groups as measured by radiostereometry. In both groups, pain and function improved substantially without any differences between groups. Patients in the early weight bearing group achieved the same result but in a shorter time.

**Interpretation** Tomofix-plate-fixated open-wedge high tibial osteotomy allows early full weight bearing without loss of correction.

## Introduction

Medial osteoarthritis of the knee can be treated with open-wedge,valgus, high tibial osteotomy (OW-HTO) (Brinkman et al. 2008). There are concerns, however, regarding the ability of the fixation technique to withstand the forces that act on the proximal tibia (Brinkman et al. 2008). Loss of correction leads to poorer results (Hernigou et al. 1987).

Ideally, the fixation technique used should be strong enough to allow early joint motion and early full weight bearing postoperatively. Initially, 15 kg of weight bearing is often allowed for 6 weeks. Depending on the fixation technique used, time to full weight bearing can take up to 3 months (Asik et al. 2006).

Angle-stable fixation plates with locking bolts have been designed for use in OW-HTO. Osteotomies fixated with these plates offer superior initial stability compared to other medial fixation plates and equal stability compared to similar plates used for CW-HTO (Gaasbeek et al. 2005, Agneskirchner et al. 2006). With these implants, immediate full weight bearing appears to be possible (Takeuchi et al. 2008, 2009).

Radiostereometric analysis (RSA) has been used to document motion at the osteotomy in OW-HTO using standard weight bearing protocols (Luites et al. 2009). The aim of this study was to measure migration at the osteotomy using RSA in patients after OW-HTO who were rehabilitated with an early full weight bearing protocol (E). The primary research question was whether there would be a difference in motion at the osteotomy because of the early full weight bearing, as measured by RSA, compared to a historical cohort of patients rehabilitated using a standard protocol (S). Secondary research questions were whether there was a difference in time to walking without aid, and improvement in pain and knee function.

## Patients and methods

After approval of the study by the institutional medical ethics committee (registration number 2005/217), we prospectively recruited patients—aged between 18 and 60 and who were scheduled for HTO—between December 2005 and December 2006. They formed the E group.

The patients in the standard weight bearing group (S) (n = 23) had previously been enrolled in a randomized controlled trail of open-wedge versus closed-wedge HTO, between December 2001 and August 2004, based on the same inclusion criteria as used in this study (Luites et al. 2009).

Both groups involved patients with medial knee osteoarthritis, with a varus deformity smaller than 12°. Exclusion criteria were rheumatoid arthritis, insufficiency of the medial collateral ligament, patella-femoral complaints, previous knee surgery, and/or a BMI of > 30. The aim was an overcorrection to a mechanical axis of 3° valgus.

**Figure 1.**
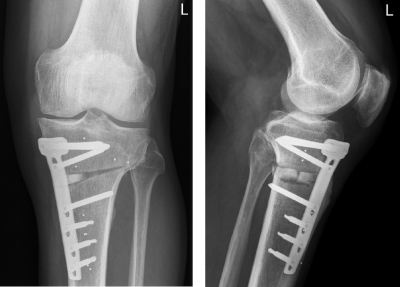
The OW-HTO, Tomofix implant and tantalum beads used for RSA.

Two experienced surgeons performed all osteotomies in both groups using standard surgical techniques. In the resultant opening, a ChronOS β-tricalcium phosphate wedge (Mathys, Bettlach, Switzerland) was inserted to facilitate bone growth. All osteotomies were fixated with the medial TomoFix plate (Figure 1) and screws (Synthes GmbH, Oberdorf, Switzerland). In the S group, a standard postoperative weight bearing protocol was used: 10–15 kg for 6 weeks and full weight bearing thereafter. The patients in the E group were allowed partial weight bearing (15 kg) until pain and wound healing permitted full weight bearing during the first 2 weeks. All patients were asked to record weight bearing in a diary to document time to full weight bearing.

**Figure 2.**
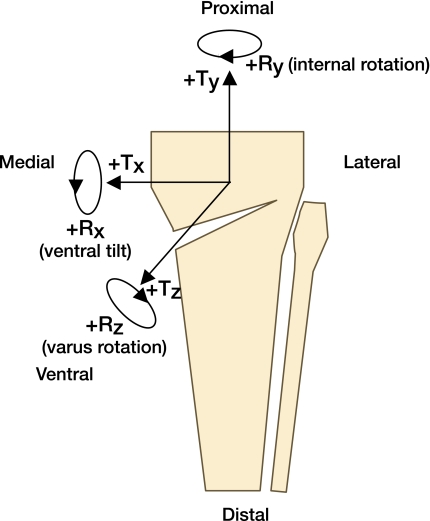
The 6 possible directions of migration are shown schematically. T: transalation in mm; R: rotation in degrees; x: transverse axis; y: longitudinal axis; z: sagittal axis.

Migration was measured using RSA; 5 to 9 tantalum beads (1.6 mm) were inserted in the proximal and distal ends of the osteotomy with a special instrument (Mathys, Bettlach, Switzerland) at the time of operation (Figure 1). Digital baseline RSA radiographs (165 d.p.i. and 11-bit gray scale resolution) were made with a digital radiology system (Agfa-Gevaert AG, Rijswijk, the Netherlands), within 1 week postoperatively after mobilization of the patient. Follow-up images were planned at 3 and 6 weeks, and 3 and 12 months postoperatively. RSA-CMS software (version 4.3.1.0 20041006 beta; Medis, Leiden, the Netherlands) was used to analyze the radiographs and calculate migration (Vrooman et al. 1998). Migration was defined as motion of the center of gravity of the proximal tibial part relative to the distal tibial part in mm, in all 3 directions and in degrees (°) around all 3 axes (Figure 2) (Valstar et al. 2005). The mean migration in all directions was calculated at all postoperative follow-up moments, as was the increase in migration during three intervals: from 0 to 6 weeks, from 6 weeks to 3 months, and from 3 months to 12 months. Movement was classified as: < 1 mm or 2° in any direction, stable, or > 1 mm or 2° in 1 or more directions, which is (possibly) clinically relevant migration.

To assess the detection limit, double examinations were done in all patients at the 6-week follow-up evaluation. These radiographs were used to calculate the upper limits of the 99% confidence interval (CI) (Mjoberg 1986). The detection limit was 0.2 mm, 0.1 mm, and 0.3 mm for the translations in the x, y, and z directions and 0.8°, 0.8°, and 0.4° for the rotations about the x, y, and z axes, respectively, which is sufficient to detect motion considered possibly clinically relevant.

Knee function was measured clinically preoperatively, at 6 weeks and at 3, 6, and 12 months postoperatively using the WOMAC, Lysholm and KOOS scores; pain was assessed on a visual analog scale (VAS). Patients documented the amount of weight bearing and use of a walking aid (crutches or cane) in a diary each day during the first 6 weeks and every week thereafter until they walked without any aid. Standard AP and lateral radiographs were taken postoperatively during the first week and after 6 and 12 months to assess bone healing.

The time until walking without a walking aid, VAS pain score, Lysholm knee function score, and motion at the osteotomy at the 6-week, 3-month, and 12-month follow-up and measurement intervals on RSA analysis from the E group were compared to the data from the S group.

### Statistics

To detect a significant difference in migration of 0.7 mm and/or 1.1° between the groups, sufficient to detect a clinically relevant difference in migration, 13 individuals in the E group would be required, according to an a priori performed power calculation with α = 0.05, power = 90%, and n = 23 in the S group. To find possible confounding factors, Student's t-test was used to detect preoperative differences in the patient characteristics between the groups. The non-parametric Wilcoxon signed rank sum test was used to detect increases in clinical scores and migration between 2 consecutive follow-up times within each group. The Mann-Whitney U test was used to compare the clinical scores, pain, and migration exceeding the detection limit between groups at different preoperative and postoperative follow-up times. Fisher's exact test was used to analyze differences between both groups in the number of patients with clinically relevant migration at the osteotomy, patients with a walking distance of less than 1 km, and patients using a walking aid. Tests were two-sided and probability values less than 0.05 were considered statistically significant. SPSS software version 12.0.1 was used to perform the statistical analyses.

## Results

14 osteotomies were performed in 14 patients (10 males) with a mean age of 49 years (SD 9), a mean weight of 81 kg (SD 11), without any complications. The mean varus angle preoperatively was 6° (SD 3). The mean angle of correction was 9° (SD 2). The preoperative patient characteristics of the early weight bearing (E) group did not differ statistically significantly from those of the standard weight bearing (S) group (Table 1). Also, the distance patients could walk preoperatively and the preoperative VAS and Lysholm scores were not statistically significantly different. Postoperatively, one complication occurred. A wound infection was diagnosed 5 weeks postoperatively and treated successfully with antibiotics and wound debridement. All osteotomies had healed at 12 months.

**Table 1.** Preoperative patient characteristics for the early weight bearing (E) group and the standard weight bearing (S) group. Mean (SD)

	E	S	p-value
Age	49 (8)	53 (8)	0.1
Weight	81 (11)	86 (12)	0.3
Height	177 (8)	176 (11)	0.4
BMI	26 (3)	28 (3)	0.1
Varus angle pre-op	6.0° (2.8°)	5.0° (2.6)	0.3
Planned correction	8.9° (2.4°)	8.1° (3.1)	0.3
VAS pain	51 (25)	56 (22)	0.6
Lysholm	57 (16)	63 (16)	0.3

The time to full weight bearing in the E group ranged between 15 and 39 days (mean 26, SD 8). Pain during walking at the moment of full weight bearing ranged from 0 to 40 (mean 21, SD 14). 2 patients walked without crutches within 3 weeks of surgery. 6 weeks postoperatively, 7 patients walked without the support of a walking aid, 6 patients walked with a cane, and the patient with the wound infection used 2 crutches. There was no association between the use of a cane and the KOOS pain score or KOOS function score at 6 weeks. At 8 weeks, 3 more patients stopped using the cane and within 3 months, none of the patients needed support. This was statistically significantly different from the S group at 3 months (p = 0.005). All the patients in the S group used crutches at the 6-week follow-up, as prescribed in the protocol. At 3 months, 12 of the 23 patients did not use crutches, and at 6 months all patients except one were able to walk without support. In the E group, 10 patients could walk a distance of more than 1 km at 3 months; this was statistically significantly more than in the S group (p = 0.04), in which 7 patients could walk more than 1 km. Pain decreased statistically significantly (p < 0.005) in all E-group patients during the first year, from a mean of 51 (SD 25) to a mean of 14 (SD 12)—a mean decrease of 38 (SD 28) points. This was not statistically significantly different (p = 0.3) compared to the improvement in the S group (mean 25, SD 28). The improvement in the Lysholm score in the E group at 6 weeks (mean 7, SD 31) was not statistically significant (p = 0.4); nor did it differ significantly from the improvement in the S group (mean 2, SD 23) (p = 0.6). At the 6-month and 12-month follow-up, the mean Lysholm score had improved statistically significantly (p = 0.009 and p = 0.003, respectively). At 12 months, the improvement in the Lysholm score in the E group (mean 19, SD 20) was not statistically different from that in the S group (mean 20, SD 14) (p = 0.9).

The overall satisfaction of the patients at the 12-month follow-up was “excellent” in 9 patients, while 4 patients scored “good”. 1 patient scored “fair”, and as joint degeneration progressed, he received a total knee arthroplasty 14 months postoperatively. 16 patients in the S group were satisfied, 5 of whom scored “good” and 5 of whom scored “excellent”. The other 9 patients scored “satisfied”; 5 patients scored “fair” and 2 patients scored “poor”. In the E group, all KOOS scores and all WOMAC scores had improved statistically significantly at the 6-month follow-up (p < 0.05) (Table 2).

**Table 2.** KOOS, WOMAC and Tegner scores preoperatively and at the postoperative follow-up times for the early weight bearing (E) group. Mean (SD)

Score	Preoperatively	6 weeks	3 months	6 months	12 months
KOOS					
Pain	43 (17)	67 (23)^a^	68 (22)^a^	78 (21)^a^	74 (19)^a^
Symptom	47 (14)	54 (12)	52 (14)	58 (11)^a^	60 (15)^a^
ADL	47 (18)	67 (18)	69 (21)^a^	77 (26)^a^	76 (19)^a^
Sp/Rec	21 (20)	20 (22)	37 (30)	47 (32)^a^	41 (27)^a^
QOL	35 (11)	36 (12)	40 (18)	44 (16)^a^	49 (11)^a^
WOMAC					
Total	49 (16)	30 (17)^a^	28 (20)^a^	21 (22)^a^	17 (14)^a^
Pain	11 (4)	6 (4)^a^	6 (5)^a^	4 (4)^a^	4 (4)^a^
Stiffness	3 (1)	2 (1)^a^	2 (1)^a^	1 (1)^a^	1 (1)^a^
Function	36 (12)	23 (12)^a^	21 (14)^a^	15 (18)^a^	16 (13)^a^
TEGNER	1.9 (1.4)			2.9 (1.6)	3.1 (1.5)
ADL: activities of daily living; Sp/Rec: sport/recreation; QOL: quality of life.					
^a^ p < 0.05 relative to the preoperative score.					

### RSA results

The mean migrations in the E group found at 3 follow-up times (Table 3) and the mean migrations found during the time intervals (Table 4) were small and within the measurement error in all directions. At 1 year, 12 patients had migration grade 1 (less than 1 mm or 2° in one or more directions) and 2 patients (14%) had grade 2 (more than 1 mm or 2° in one or more directions). At any of the follow-up times, there were no statistically significant differences between groups in the number of osteotomies that migrated more than what was considered clinically significant (grade 2). In both grade-2 cases of the E group, the only possibly clinically relevant migration was rotation in the dorsal direction around the x-axis (posterior tilting). Both had different migration patterns during the follow-up period, which resulted in a posterior tilt of over 2° at 1 year. In one case, the posterior tilt was 0.3° at 3 weeks, then increased to 1.1° at 6 weeks, and to 2.1° at 3 months; thereafter, it stabilized. This patient started full weight bearing at day 34 and was using a cane until week 11. In the other case, the posterior tilt was 1.0° in the first 3 weeks, increasing gradually to 1.2° at 6 weeks and 1.5° at 3 months, and with a further increase to 2.3° at 1 year. This patient started full weight bearing at day 31 and was using a cane until week 12.

**Table 3.** Migration in the early weight bearing (E) group and the standard weight bearing (S) group at 3 follow-up times. Values are translation (T) in mm and rotation (R) in degree.

Follow-up	E		S	
Axis	Mean (SD)	(Range)	Mean (SD)	(Range)
6 weeks				
Tx	-0.02 (0.36)	(-0.70–0.60)	0.17 (0.24) **^b^**	(-0.09–0.84)
Ty	-0.10 (0.24)	(-0.75–0.27)	-0.15 (0.19) **^b^**	(-0.64–0.19)
Tz	-0.01 (0.14) **^c^**	(-0.20–0.30)	-0.19 (0.37) **^b, c^**	(-0.79–0.46)
Rx	-0.16 (0.74)	(-1.18–1.78)	-0.57 (0.89) **^b^**	(-3.20–1.03)
Ry	-0.16 (0.52)	(-0.92–0.60)	-0.15 (0.60)	(-1.70–0.80)
Rz	-0.01 (0.67)	(-1.69–1.04)	-0.05 (0.66)	(-1.79–1.10)
3 months				
Tx	-0.01 (0.40)	(-0.86–0.69)	0.14 (0.24) **^b^**	(-0.27–0.71)
Ty	-0.13 (0.22)	(-0.75–0.19)	-0.17 (0.25) **^b^**	(-0.74–0.22)
Tz	-0.05 (0.20)	(-0.50–0.32)	-0.17 (0.33) **^b^**	(-0.78–0.40)
Rx	-0.41 (0.99)	(-2.11–2.17)	-0.88 (1.47) **^b^**	(-6.14–1.49)
Ry	-0.09 (0.58)	(-1.18–0.94)	-0.05 (0.47)	(-0.84–0.73)
Rz	0.06 (0.75)	(-1.72–1.42)	0.16 (0.54)	(-0.79–1.04)
12 month				
Tx	-0.03 (0.40)	(-0.81–0.73)	0.07 (0.25)	(-0.43–0.58)
Ty	-0.18 (0.27) **^a^**	(-0.97–0.16)	-0.29 (0.49) **^b^**	(-1.83–0.14)
Tz	-0.08 (0.21)	(-0.53–0.35)	-0.23 (0.32) **^b^**	(-0.76–0.53)
Rx	-0.73 (0.88) **^a^**	(-2.26–0.60)	-1.18 (1.12) **^b^**	(-4.45–0.73)
Ry	-0.07 (0.51)	(-0.89–0.75)	0.12 (0.67)	(-0.78–2.06)
Rz	0.19 (0.82)	(-1.76–1.46)	0.45 (0.86)	(-0.68–2.06)
In 1 patient in the S group, the postoperative RSA radiograph was missing; for this patient, the 6-week result served as baseline.				
**^a,b^** Significant migration (2-sided, p < 0.05) at the follow-up within the **^a^** E and **^b^** S groups, tested with the Wilcoxon test, but not clinically relevant.				
**^c^** Significant difference in migration (2-sided, p < 0.05) in the S group compared with the E group, tested with the Mann–Whitney U test, but migrations were below the detection limit.				

**Table 4.** Increases in migration in the early weight bearing (E) group and the standard weight bearing (S) group from 6 weeks to 3 months and from 3 to 12 months postoperatively. Values are translation (T) in mm and rotation (R) in degree.

	E		S	
	1.5–3 months	3–12 months	1.5–3 months	3–12 months
Tx	0.01 (0.08)	-0.02 (0.09)	-0.03 (0.23)	-0.07 (0.21)
Ty	-0.04 (0.06) **^a^**	-0.04 (0.07) **^a^**	-0.02 (0.24)	-0.13 (0.37)
Tz	-0.07 (0.17)	-0.02 (0.11)	0.02 (0.20)	-0.06 (0.23)
Rx	-0.25 (0.35) **^a^**	-0.30 (0.47) **^a^**	-0.29 (0.82)	-0.40 (0.89)
Ry	0.07 (0.48)	0.02 (0.25)	0.06 (0.45)	0.21 (0.55)
Rz	0.07 (0.20)	0.12 (0.18) **^a^**	0.10 (0.39)	0.29 (0.55) **^b^**
**^a, b^** Significant increase (2-sided, p < 0.05) during the time intervals within the **^a^** E and **^b^** S groups, tested with the Wilcoxon test, but not clinically relevant.				

The RSA data of the E group were compared to the RSA data of the S group (Tables 3 and 4). In the S group of 23 patients, 7 cases showed grade-2 migrations at 1 year. In 1 of these 7, the lateral cortex of the tibia fractured intraoperatively. During the first 6 weeks of partial weight bearing, the proximal tibial part tilted 3.0° posteriorly. This posterior tilt increased between 6 weeks and 3 months to 6.1°. After that, it rotated in the opposite direction. At 12 months, this had resulted in a migrated position of 4.4° posterior tilt, a varus rotation of 2°, and 2 mm of posterior translation. In 4 osteotomies, migrations gradually increased until 6 months, with small increases in the second half-year, resulting in a posterior tilt ranging between 2.0 and 2.4. 3 of the patients only stopped using crutches between 3 and 6 months postoperatively. 2 cases showed migration, which gradually increased during the first year, resulting in varus rotation (2.1° and 2.0°) around the z-axis. One patient who stopped using crutches between 3 and 6 months postoperatively also had 2.1° of medial rotation around the y-axis. As in the E group, the 7 grade-2 cases in the S group did not migrate more than what was considered possibly clinically significant in the second half-year, and were considered stable at 1 year.

There were no statistically significant differences in mean migration between groups at any of the follow-up times for RSA measurement or time intervals between the follow-ups.

## Discussion

In this series of OW-HTO patients, full weight bearing was allowed as soon as wound healing and pain permitted it, but no loss of correction and no adverse effects occurred. There was no difference in motion at the osteotomy between groups at any of the time points, as measured by RSA. At the 1-year follow-up in the E group, varus rotation—which might influence leg alignment—was small (mean 0.2°, SD 0.8) and posterior tilt—which might influence slope—was also small (mean 0.7°, SD 0.9).

Because of the early weight bearing, patients regained knee function in a shorter time. Pain and knee function scores were not, however, influenced by the weight bearing protocol; in both groups, they improved statistically significantly and were nearly equal at 1 year.

In HTO, the implant used for fixation should be able to retain the correction achieved at the time of surgery. RSA has been used in vitro as well as in clinical studies to measure initial fixation stability and postoperative stability at specific intervals after HTO (Gaasbeek et al. 2005, Luites et al. 2009, Magyar et al. 1999, Pape et al. 2004). Using RSA in a cadaver model comparing initial stability in OW and CW using medial and lateral angle stable plates for fixation, Gaasbeek et al. (2005) found no difference in motion at the osteotomy. Similarly, in a prospective randomized clinical trial comparing stability in CW and OW-HTO fixated with TomoFix implants, Luites et al. (2009) found no difference in initial stability and ability to retain the correction at 1-year follow-up. In our series, no differences in motion (measured by RSA at the osteotomy) were seen between groups. No relation between the amount of weight bearing or the use of crutches and migration was found.

Rehabilitation protocols and time to full weight bearing after HTO vary. The amount of initial weight bearing that is allowed postoperatively depends strongly on the type of fixation used. Noyes et al. (2006) reported that full weight bearing was possible after 8 weeks in OW-HTO fixated with a Puddu plate. In their series, rigid autologous grafts were used to increase initial stability and a long leg brace was prescribed for the first 8 weeks. Also using a Puddu plate for fixation together with iliac crest autografts or frozen allografts and a knee-hinged immobilizer to increase stability, Asik et al. (2006) reported that full weight bearing was possible after 3 months. Lobenhoffer et al. (2004) and Staubli et al. (2003), using Tomofix for fixation in OW-HTO without filling the gap and without using a brace or cast, found that full weight bearing was possible at 8 weeks and 10 weeks, respectively. More recently, a shorter time to full weight bearing after OW-HTO has been reported; in a series of 57 OW-HTOs again using Tomofix for fixation, Takeuchi et al. (2009) allowed full weight bearing after 2 weeks. As with our findings, they did not observe any loss of correction or implant failure. Patients in Takeuchi's series were older on average: 69 years as compared to 49 years in this series.

Gap filling in OW-HTO may be used to increase initial stability and facilitate or promote bone healing. Takeuchi et al. (2009) used tri-calcium phosphate (β-TCP) and hydroxyapatite wedges to help distribute the stress at the osteotomy across the wedge and the plate. No mention was made of bone healing time or time to full bone remodeling of the porous bone substitutes; however, no non-unions were reported. The porous β-TCP bone substitute inserted into the gaps created in our patients in the E and S groups is not shaped to fill the gap, and does not provide added initial stability. No non-unions were seen in either group, and bone healing was complete in all patients at the 1-year follow-up. Furthermore, full resorption of bone substitute was found to be present in almost all patients, as has been described in a previous histological analysis on the resorption of β-TCP in OW-HTO (van Hemert et al. 2004). Lobenhoffer et al. (2004) and Staubli et al. (2003) did not use grafts or bone substitutes to fill the gap, and reported that full remodeling of the medial cortex can take up to 1 year. They reported 2 cases of non-union in 262 patients and 1 non-union and two re-varisations in 92 patients, respectively. It can be concluded that bone healing occurs with or without filling of the gap. Furthermore, bone healing times do not vary. It can therefore be debated whether the gap should be filled at all. We conclude that fixation stability in our series of patients was solely dependent on the stability provided by the Tomofix plate; this did not lead to delayed healing or loss of correction.

Although early weight bearing appears to be safe in OW-HTO fixated with Tomofix plates, other factors influence the actual time at which patients start full weight bearing. Remarkably, the use of a walking aid was not related to pain or the function score at that time; patients in the E group did not experience more pain because of the early weight bearing, and their function score did not improve faster. In this group patients did have a shorter period of time to full weight bearing; furthermore, their walking distances increased more quickly. Although the patients in the E group had a shorter time to full weight bearing and were walking longer distances earlier in their recovery, this was not reflected in their Lysholm scores.

Criticisms that can be leveled at this study include the small sample size of the E cohort and the fact that the patients were from different cohorts of OW-HTO. They were from the same patient population, however, and patients in both groups were recruited based on the same criteria. Inclusion criteria were stringent to minimize the influence of factors other than the amount of weight bearing on the primary outcome measurement, i.e. motion at the osteotomy. Demographics were similar. Furthermore, all patients in both groups were operated on by the same surgeons using standardized technique.
